# Estimation of Vertical Ground Reaction Forces and Sagittal Knee Kinematics During Running Using Three Inertial Sensors

**DOI:** 10.3389/fphys.2018.00218

**Published:** 2018-03-22

**Authors:** Frank J. Wouda, Matteo Giuberti, Giovanni Bellusci, Erik Maartens, Jasper Reenalda, Bert-Jan F. van Beijnum, Peter H. Veltink

**Affiliations:** ^1^Institute for Biomedical Technology and Technical Medicine (MIRA), University of Twente, Enschede, Netherlands; ^2^Xsens Technologies B.V., Enschede, Netherlands; ^3^Roessingh Research and Development, Roessingh Rehabilitation Hospital, Enschede, Netherlands; ^4^Centre for Telematics and Information Technology, University of Twente, Enschede, Netherlands

**Keywords:** machine learning, artificial neural networks, reduced sensor set, inertial motion capture, running, kinetics

## Abstract

Analysis of running mechanics has traditionally been limited to a gait laboratory using either force plates or an instrumented treadmill in combination with a full-body optical motion capture system. With the introduction of inertial motion capture systems, it becomes possible to measure kinematics in any environment. However, kinetic information could not be provided with such technology. Furthermore, numerous body-worn sensors are required for a full-body motion analysis. The aim of this study is to examine the validity of a method to estimate sagittal knee joint angles and vertical ground reaction forces during running using an ambulatory minimal body-worn sensor setup. Two concatenated artificial neural networks were trained (using data from eight healthy subjects) to estimate the kinematics and kinetics of the runners. The first artificial neural network maps the information (orientation and acceleration) of three inertial sensors (placed at the lower legs and pelvis) to lower-body joint angles. The estimated joint angles in combination with measured vertical accelerations are input to a second artificial neural network that estimates vertical ground reaction forces. To validate our approach, estimated joint angles were compared to both inertial and optical references, while kinetic output was compared to measured vertical ground reaction forces from an instrumented treadmill. Performance was evaluated using two scenarios: training and evaluating on a single subject and training on multiple subjects and evaluating on a different subject. The estimated kinematics and kinetics of most subjects show excellent agreement (ρ>0.99) with the reference, for single subject training. Knee flexion/extension angles are estimated with a mean RMSE <5°. Ground reaction forces are estimated with a mean RMSE < 0.27 BW. Additionaly, peak vertical ground reaction force, loading rate and maximal knee flexion during stance were compared, however, no significant differences were found. With multiple subject training the accuracy of estimating discrete and continuous outcomes decreases, however, good agreement (ρ > 0.9) is still achieved for seven of the eight different evaluated subjects. The performance of multiple subject learning depends on the diversity in the training dataset, as differences in accuracy were found for the different evaluated subjects.

## 1. Introduction

Running is a very popular form of physical activity, that is often accompanied with a high occurrence of lower extremity injuries (incidence rate varies between 19.4 and 79.3%; van Gent et al., 2007). It is assumed that there is a correlation between the development of these injuries and a runner's technique (Goss et al., 2012). Additionally, improvements in running technique could lead to improved running performance (Kyröläinen et al., 2001; Tartaruga et al., 2012; Folland et al., 2017). Identifying the parameters in running technique that might be associated with injury development and/or running performance improvement requires a biomechanical analysis. This has traditionally been performed inside a gait laboratory using a three-dimensional optical motion capture system and force plates (Novacheck, 1998). The most relevant kinematic and kinetic parameters analyzed are: joint angles (Devita and Skelly, 1992; Edwards et al., 2012) and ground reaction forces (Cavanagh and Lafortune, 1980), as these are important determinants of running technique (Goss et al., 2012). Discrete kinetic parameters that are related to running injuries and/or performance are: loading rate and peak vertical ground reaction forces (Crowell and Davis, 2011; Goss et al., 2012; Schmitz et al., 2014), whereas maximal knee flexion during stance is a relevant discrete kinematic parameter (Edwards et al., 2012). However, a lab setting is not identical to the regular running environment and may therefore result in different kinematics and kinetics (Sinclair et al., 2013). Previous studies have confirmed this, showing significant differences between running on a treadmill and outdoors (Nigg et al., 1995). Furthermore, dissimilarities in running kinematics can also occur as a result of force plate targeting in overground lab running (Challis, 2001). Therefore, a system capable of measuring relevant parameters outside of a laboratory may address these shortcomings.

Kinematic analysis can be performed in an ambulatory setting using inertial measurement units (IMUs) (see for instance, Roetenberg et al., 2013). Reenalda et al. (2016) have used IMUs to measure the effects of fatigue on running mechanics during an actual marathon. However, this approach requires one sensor to be attached on each main body segment along a continuous “kinematic chain,” and therefore results in a large number of sensors and extensive subject preparation. Data driven approaches were shown to have potential for reducing the number of sensors in motion capture. Tautges et al. (2011) proposed a method for full-body motion capture by using a limited number of accelerometers; however, their nearest neighbor approach requires a database of prerecorded movements to be available at run-time. Wouda et al. (2016) showed comparable performance with a reduced sensor setup using an artificial neural network (ANN), trained to map five orientations to a full-body pose. ANNs have the advantage to create a “model” for mapping certain inputs to outputs based on the dataset used for training (Alpaydin, 2009). Running applications using a minimal inertial sensor set have mainly focused on temporal outcomes, such as the use of gyroscopes on the feet to estimate temporal running parameters (McGrath et al., 2012). Bailey and Harle (2014, 2015) showed that with foot-mounted IMUs this can be extended to estimate spatio-temporal running parameters.

Ground reaction forces are also relevant outcome parameters for running analysis (e.g., Cavanagh and Lafortune, 1980; Novacheck, 1998; Riley et al., 2008; Caekenberghe et al., 2013; Clark et al., 2014), since abnormal peak and/or loading rate values can lead to impact and overuse injuries, when the stress/frequency combination is above the runner's threshold (Hreljac, 2004; Milner et al., 2007). However, none of the aforementioned approaches provided users with kinetic information. Efforts to move kinetic analyses out of the laboratory setting have proven to be effective for trunk bending (Faber et al., 2016), gait (Karatsidis et al., 2017), dance (Shippen and May, 2012), and running (Pavei et al., 2017). However, aforementioned approaches require full-body kinematic information. The peak vertical ground reaction forces (vGRF) estimation approach of Charry et al. (2013) relied only on tibial accelerations, but was not suitable for estimation of kinetics during the whole stance phase. An approach relying only on trunk accelerations was not sufficient for vGRF estimation using a mass-spring-damper model (Nedergaard et al., 2017).

To the best of our knowledge, there is no system that can provide runners with insights in both their kinematics and kinetics in an outdoor setting. The aim of this study is to assess the validity of a method to estimate knee joint angles and vertical ground reaction forces during running using an ambulatory minimal body-worn sensor setup. An ANN is trained to estimate joint angles based on lower leg orientations relative to the pelvis, similar to the approach presented in previous work (Wouda et al., 2016). Corresponding performance is evaluated using both inertial and optical full-body motion capture data. The estimated joint angles in combination with sensor accelerations can be fed into a second ANN which estimates (vertical) ground reaction forces. The proposed method was evaluated using continuous outcomes (vGRF and knee angle profiles) and discrete outcomes (peak vGRF, loading rate, and maximal knee flexion during stance). The findings of this study could have potential for future applications in prevention of running injuries and improvement of running performance.

## 2. Materials and methods

### 2.1. Measurement protocol

Eight healthy experienced runners (8 males; age: 25.1 ± 5.2 years; height: 183.7 ± 4.5 cm; weight: 77.7 ± 9.4 kg; body mass index: 23.0 ± 2.5 kg/m^2^) voluntarily participated in this research. The runners were recruited from a local track and field club and all reported no recent injuries. Subjects were instructed to run at 3 different speeds (10, 12, and 14 km/h, in this order) for 3 min each on an instrumented treadmill, located at the gait laboratory of the Roessingh Research and Development (Enschede, the Netherlands). A warm-up session at a self-selected running speed (of approximately 3 min) was performed by all subjects preceding the measurements. The ethics committee of the Faculty of Electrical Engineering, Mathematics and Computer Science at the University of Twente approved this protocol and all subjects provided written informed consent prior to the measurements.

### 2.2. Measurement setup

**Figure 1:**
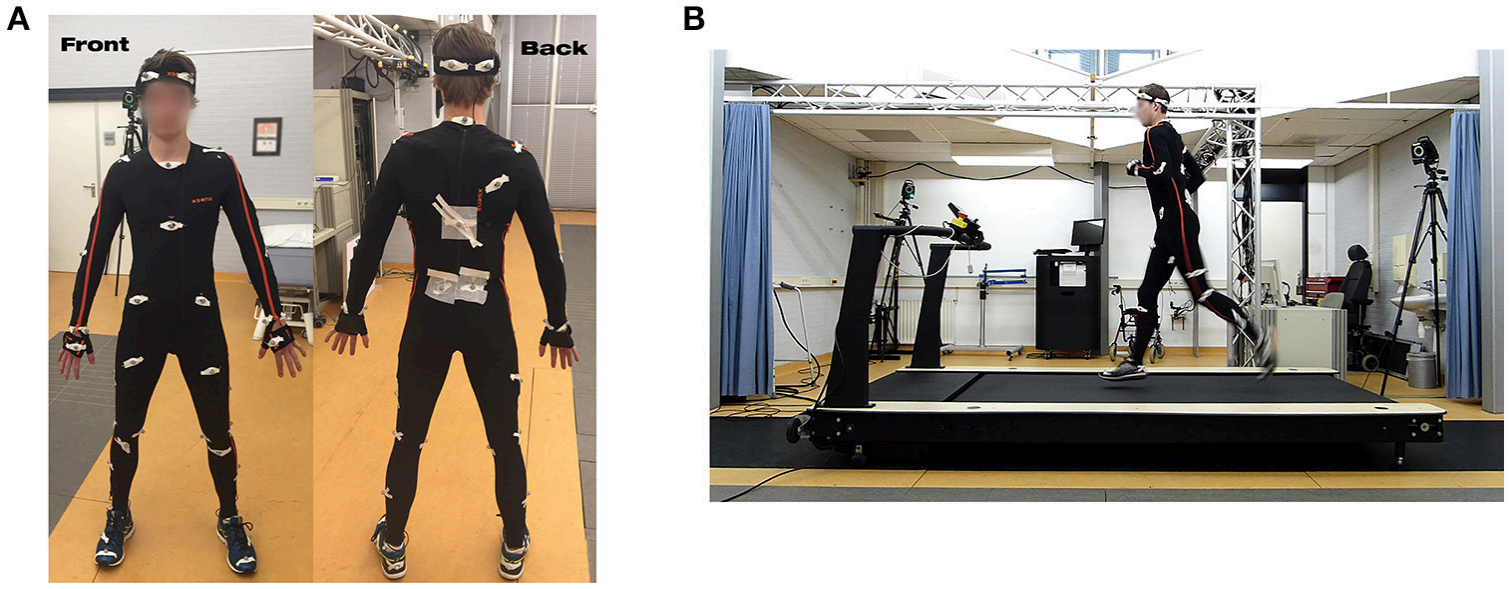
The measurement setup, **(A)** shows a front and back view of the sensor and retroreflective marker placement **(B)** shows the measurement setup (only 2 cameras are visible in this angle). Subjects wore a Lycra suit to hold the IMUs in place, which was customized with holes to accommodate the placement of retroreflective markers on the subject's skin. In this manner it was possible to measure kinematics simultaneously using both an inertial and optical motion capture system. The retroreflective markers were placed according to the Plug-in Gait protocol. To ensure retroreflective marker placement during the whole measurements, tapes were placed around these markers. Note that written informed consent was provided for use of these images.

Reference kinematics were recorded with an optical motion capture system using the Plug-in Gait protocol^1^ (Nexus 1.8.5, Vicon, Oxford, UK), with 41 retroreflective markers placed directly on the runners' skin, as shown in Figure 1. The position of these markers was captured (at 100 Hz) by six high-speed infrared cameras (MX-13, Vicon, Oxford, UK) placed around the treadmill. Any object that could block the camera view or produce undesired reflections was removed from the measurement environment. Additionally, kinematics were synchronously captured using the Xsens MVN Link inertial motion capture system (Xsens, Enschede, the Netherlands), consisting of 17 IMUs placed at both shoulders, upper arms, lower arms, hands, upper legs, lower legs, feet, head, sternum, and pelvis (Roetenberg et al., 2013). The required full-body Lycra suit (for IMU placement) was modified with holes to reduce motion artifacts of the retroreflective markers, which are placed directly on the subject's skin. Full-body kinematics were exported using the accompanying software (MVN studio 4.3.7, Xsens, Enschede, Netherlands) at a selected sampling frequency of 240 Hz. Subjects ran on a S-Mill instrumented treadmill (ForceLink, Culemborg, the Netherlands), with a running area of 250 × 100 cm, which can be seen in Figure 1. The treadmill was equipped with a 1-dimensional force plate, able to measure reference vGRF at 1,000 Hz. Data of the different systems were synchronized using an analog synchronization signal.

### 2.3. Data processing

The different trials were cropped to contain only kinematic and kinetic data of running at a steady speed, i.e., starting and stopping of the treadmill was disregarded. Optical kinematic data was processed using Plug-in Gait (Kadaba et al., 1990; Davis et al., 1991). The optical and inertial motion data did not require coordinate systems alignment as the outcome measures were expressed in the joint frame, according to ISB conventions (Wu et al., 2002). The vGRFs were low-pass filtered at 20 Hz using a zero-phase 6th order Butterworth filter, to remove noise artifacts such as vibrations of the treadmill (Sloot et al., 2015), while neither the optical nor inertial motion capture data were filtered. Beside the temporal alignment (achieved with an analog synchronization signal), the data were resampled at 120 Hz using linear interpolation (for the optical data) and downsampling (for the inertial and vGRF data), such that all synchronized data can be used in the proposed machine learning approach. This data resampling does not significantly influence the measured kinematics and kinetics, as was also concluded by Pavei et al. (2017). For analysis, the kinematic and kinetic data were segmented in stance phases using a 20 N threshold (Milner and Paquette, 2015). All data processing and statistical analyses was done in MATLAB R2017a (Mathworks, Inc., Natick, MA, USA).

### 2.4. Learning approach

**Figure 2:**
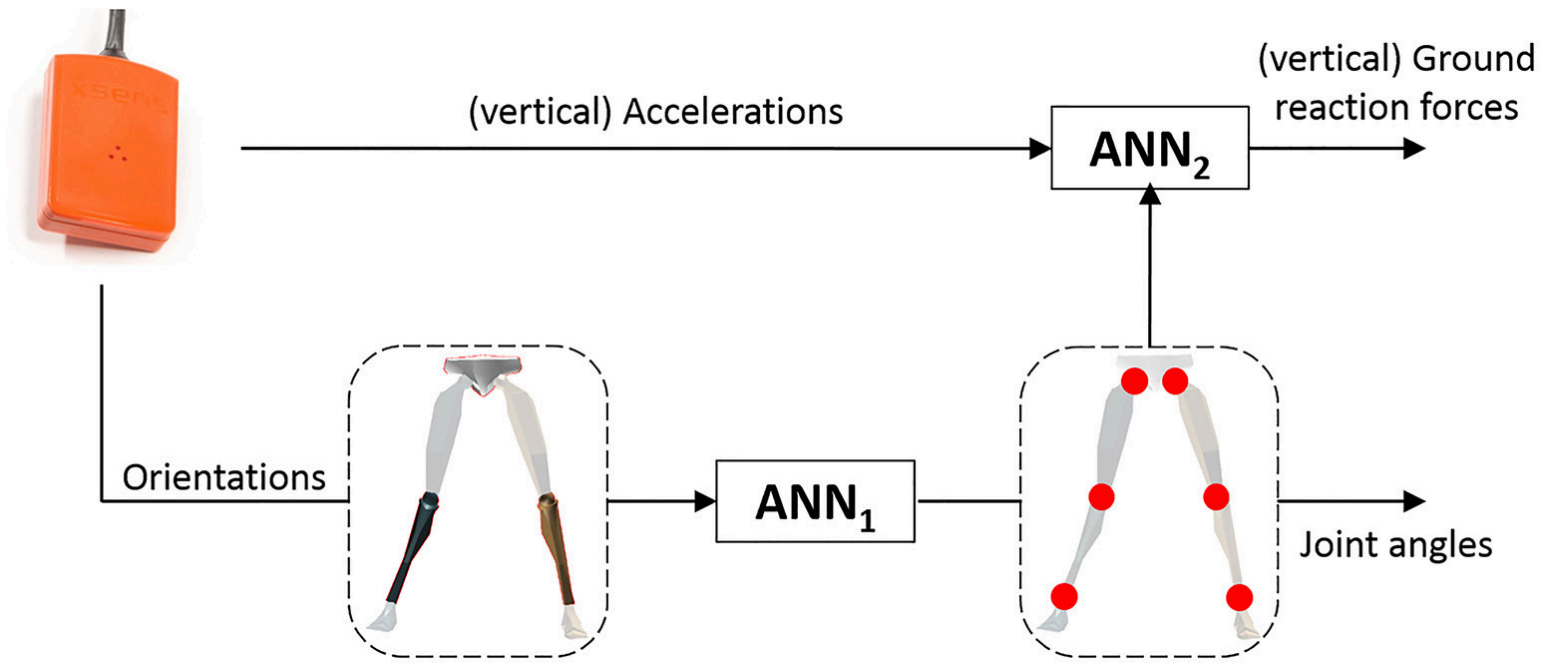
The IMU in the top left represents the sensors strapped to the lower legs and pelvis. Information from these sensors is used by two concatenated Artificial Neural Networks (ANNs) to estimate kinematics and kinetics. ANN_1_ maps the relative orientations of the lower legs (with respect to the pelvis) to lower body joint angles (hip, knee and ankle). ANN_2_ is trained to map the estimated kinematics in combination with the vertical (after transformation to the global frame) sensor accelerations to the reference ground reaction forces.

The proposed learning approach relies on data from three body-worn sensors (placed at the pelvis and lower legs), which are fed to a concatenation of two ANNs, as schematically represented in Figure 2. The first artificial neural network (ANN_1_) maps relative (to the pelvis) orientations (in quaternions) of the lower legs to joints angles, whereas the second artificial neural network (ANN_2_) maps the estimated joint angles in combination with vertical sensor accelerations (in the global frame) to vertical ground reaction forces. This architecture was chosen to allow for independent training of the two ANNs. Additionally, the proposed architecture separates the learning problems allowing for “selective” re-training of the ANNs (for instance, additional running environments can be included in the dataset of ANN_1_ without measuring GRFs simultaneously).

Estimated kinematic outputs were being compared to measured reference kinematics, which were obtained from both inertial or optical motion capture systems. To that end, two training schemes were evaluated, as shown in Table 1, to test the proposed method irrespective of the motion capture technology.

**Table 1:** The training and testing schemes for both the kinematic and kinetic estimations are represented.

**ANN**_**1**_			
**Scheme**	**Training input**	**Training output**	**Reference**
1	3 IMU orientations	IMU lower-body joint angles	IMU lower-body joint angles
2	3 IMU orientations	Plug-In Gait lower-body joint angles	Plug-In Gait lower-body joint angles
**ANN_2_**			
1	IMU lower-body joint angles	vGRF (FP)	vGRF (FP)
2	Plug-In Gait lower-body joint angles	vGRF (FP)	vGRF (FP)

*Where input to ANN_1_ is in all cases the measured relative orientations from the three on-body IMUs (placed at the pelvis and both lower legs), and the output can be from the inertial (IMU) or optical (Plug-In Gait) measurements. This is then input to the kinetic estimation part (ANN_2_), for which the output is all cases the measured vertical Ground Reaction Forces (vGRF) using the forceplates (FP)*.

Previous studies have achieved varying performance in GRF estimation (Shippen and May, 2012; Charry et al., 2013; Faber et al., 2016; Karatsidis et al., 2017; Nedergaard et al., 2017; Pavei et al., 2017). Therefore, several ANNs were trained using combinations of different input features (joint angles, pelvis, and lower leg vertical accelerations) to select the best set of input features. The selection of these input features is based on their physical relation to the ground reaction forces, where joint angles define the continuous kinematic chain (Faber et al., 2016; Karatsidis et al., 2017) and accelerations are related to force according to Newton's second law of motion.

In accordance with previous work of the authors (Wouda et al., 2016), a two-layer (with 250 and 100 neurons) function fitting neural network architecture was used for both ANNs, capable of mapping non-linearities between input and output. The networks were trained for 2,000 iterations and training was stopped early if the gradient did not decrease for 6 consecutive iterations or if the gradient was smaller than 1 × 10^−6^. The neural network toolbox of MATLAB R2017a (Mathworks, Inc., Natick, MA, USA) was used to design, train, and evaluate the ANNs described above.

Two different evaluation scenarios were evaluated to show single (section 3.1) and multiple subject (section 3.2) performance:

1. For each subject, evaluation was done using all running data at 12 km/h, while data with other speeds (i.e., 10 and 14 km/h) are used for training.
2. All data from one subject were used at turn for evaluation, while all data of remaining subjects were used for training. Note that, for the sake of simplicity, we will show only results corresponding to data of running at 12 km/h.

Scenario 1 would require every new user to perform a training phase. Scenario 2 could potentially produce a more generic model, although the lack of personalization of the network may result in decreased performance.

### 2.5. Outcome measures

The performance of the proposed method was evaluated by comparing both discrete and continuous outcomes, as commonly done in similar works about biomechanical analysis of running (Cavanagh and Lafortune, 1980; Devita and Skelly, 1992; Crowell and Davis, 2011; Edwards et al., 2012; Schmitz et al., 2014). For the knee flexion/extension (F/E) the similarity between the estimates and reference was calculated using the Pearson's correlation coefficient (ρ) and Root Mean Squared Error (RMSE) (as defined by Ren et al., 2008). The mean ρ over these different strides was calculated using a Fisher transformation to obtain a more representative average Pearson's correlation coefficient (Corey et al., 1998). Additionally, the maximum knee F/E angle during the stance phase was evaluated using a paired *t*-test (significance level of 0.05) and Bland-Altman plot (Bland and Altman, 1986). Estimated vGRFs (normalized to body weight, BW) were also evaluated using both continuous (ρ and RMSE) and discrete metrics (loading rate and peak vGRF). The kinetic analysis was however limited to the stance phase of each leg (as there is no contact during swing phase). Since the passive vGRF peak is not clearly defined for mid- or forefoot strikers, this event was determined using the peak acceleration from the lower leg IMUs (Willy et al., 2008). Using this event the loading rate was calculated as the slope of vGRF between 20 and 80 percent of the passive vGRF peak time (Willy et al., 2008; Crowell and Davis, 2011).

## 3. Results

Section 3.1 shows performance of the proposed method for training and evaluating on a single subject, where the difference between both sets is the running speed (scenario 1). Section 3.2 is about generalization of this approach over different subjects (scenario 2).

### 3.1. Single subject learning

#### 3.1.1. Kinematics estimation

The accuracy of estimated knee F/E angles based on different references (full-body IMU motion capture system or optical Plug-In Gait output) is presented in Table 2. The estimates provided by most individually trained ANNs have excellent agreement (ρ > 0.99) with the reference joint angles. Furthermore, only subject eight shows significant differences in performance between the different references.

**Table 2:** Accuracy of estimated knee flexion/extension (F/E) angles (using ANN_1_) with different training outputs (namely: IMU or Plug-in Gait-based), using single subject training and evaluation.

**Subjects**	**IMU**				**Plug-in Gait**			
	**Left F/E**		**Right F/E**		**Left F/E**		**Right F/E**	
	**ρ**	**RMSE (σ)**	**ρ**	**RMSE (σ)**	**ρ**	**RMSE (σ)**	**ρ**	**RMSE (σ)**
S01	0.99	3.24 (1.53)	0.99	4.38 (1.71)	0.99	3.56 (0.97)	0.99	4.76 (1.46)
S02	0.99	1.74 (0.48)	0.99	1.77 (0.54)	0.99	4.14 (1.39)	0.99	3.79 (1.41)
S03	0.99	2.65 (0.64)	0.99	2.05 (0.53)	0.99	3.70 (1.22)	0.99	2.58 (0.72)
S04	0.99	2.60 (0.47)	0.99	2.26 (0.58)	0.99	3.02 (1.28)	0.99	3.59 (1.41)
S05	0.99	3.39 (1.79)	0.99	3.55 (2.05)	0.99	4.03 (1.19)	0.99	4.49 (1.33)
S06	0.99	3.57 (0.67)	0.99	3.52 (0.64)	0.99	2.62 (0.54)	0.99	2.27 (0.63)
S07	0.99	3.30 (0.57)	0.99	2.86 (0.51)	0.99	5.27 (1.14)	0.99	5.41 (1.21)
S08	0.99	3.95 (1.70)	0.99	3.17 (1.49)	0.98	7.33 (2.68)	0.98	8.41 (3.02)

*Pearson's correlation coefficient (ρ) is calculated for each stride and averaged over approximately 200 strides for each subject (S01, S02, S03, S04, S05, S06, S07, and S08). The Root Mean Squared Error (RMSE) is calculated similarly over all strides. Training of the artificial neural networks was performed using running data at 10 and 14 km/h, while 12 km/h running data was used for evaluation*.

**Figure 3:**
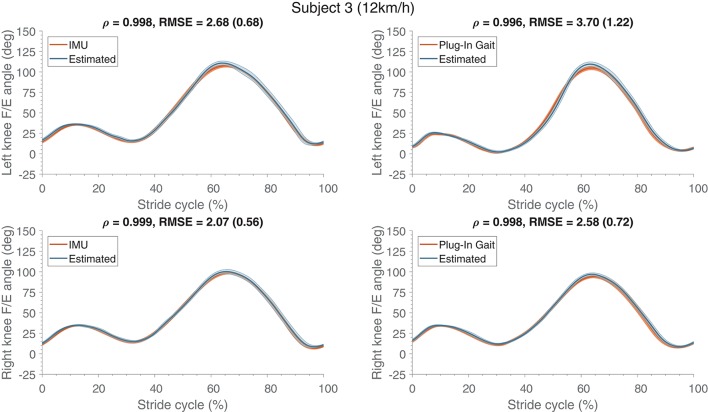
Mean (and standard deviation band) of the flexion/extension knee joint angle (in degrees) estimates are presented (normalized to the stride cycle) compared to their respective references (IMU and Plug-In Gait output). These estimates were obtained from training (using running data at 10 and 14 km/h) and evaluating (using running data at 12 km/h) on a single subject, similar results were obtained for the other subjects. The top row shows the angles of the left side and the bottom row presents the right side. At the top of each graph Pearson's correlation coefficient, root mean square error (RMSE) and the standard deviation (between the brackets) are specified, which were calculated for the estimate compared to its respective reference kinematics.

Mean (and standard deviation) of the estimated knee F/E angle profiles are shown in Figure 3 for a representative subject (S03). The largest difference between the estimate and its respective reference can be seen at the largest flexion angle, which is overestimated in all cases. As observed before in Table 2, differences between the estimates based on the various references are limited (4° on average).

**Figure 4:**
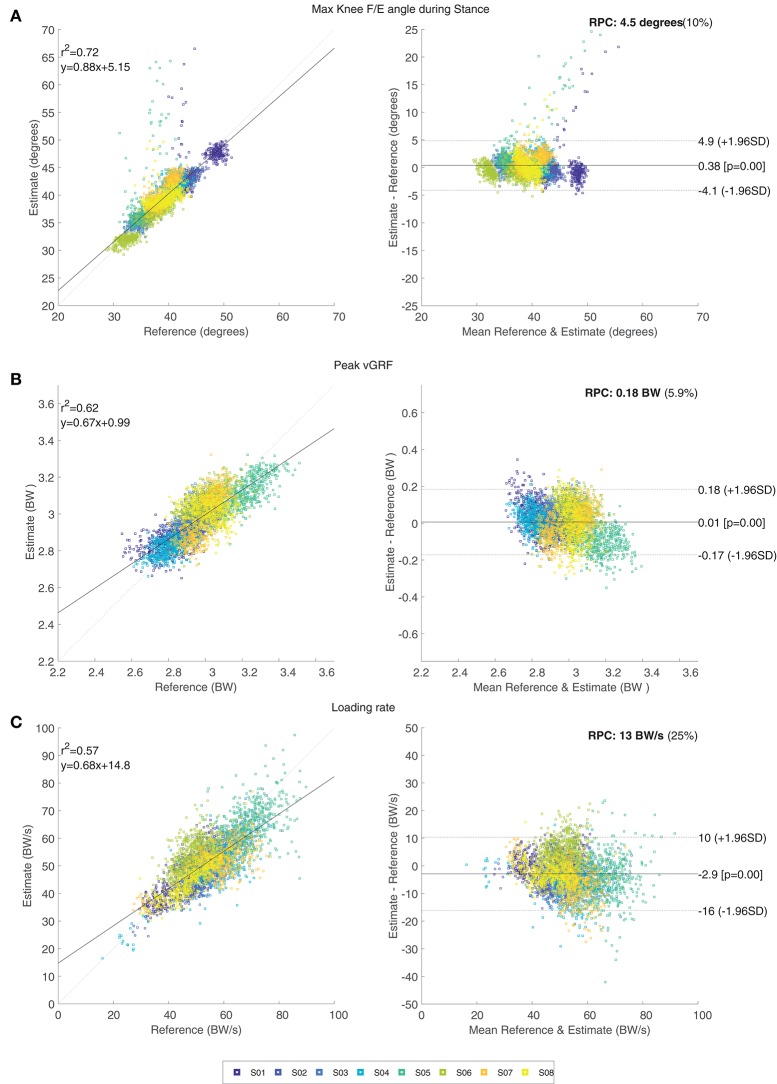
The left side shows the correlation plot of the discrete outcome measures: maximal knee flexion angle during stance **(A)**, peak vGRF **(B)**, and loading rate **(C)**. The right side shows the corresponding difference plots of those three discrete outcome measures. Approximately 4,000 data points are shown, where different subjects are represented by the various colors.

Table 3 shows the mean (and standard deviation) of the maximal knee F/E angle for each subject. Only inertial results and the corresponding estimates are presented in this table for conciseness. The mean difference in maximal knee flexion angle during stance between the estimate and its reference are < 2° for all subjects, and this result shows no significant differences (*p* > 0.05). A small bias of 0.4° was found with limits of agreement –4.1 to 4.9° for the comparison between the estimated maximal knee F/E angle during stance and the corresponding reference. Figure 4A shows the related Bland-Altman plot. Occasional outliers (for three of the evaluated subjects) can be observed, which are mostly overestimating the maximal knee F/E angle during stance.

**Table 3:** The mean (and standard deviation) of discrete outcome measures for both the estimate and its corresponding reference (based on inertial full-body motion capture data) of all subjects.

**Subjects**	**Max knee F/E angle (degrees)**		**vGRF peak (BW)**		**Loading rate (BW/s)**	
	**Reference**	**Estimate**	**Reference**	**Estimate**	**Reference**	**Estimate**
S01	45.41 (3.56)	45.05 (3.94)	2.79 (0.08)	2.83 (0.06)	44.39 (7.37)	45.52 (8.05)
S02	42.96 (1.55)	42.56 (1.29)	2.96 (0.07)	2.94 (0.05)	50.72 (4.93)	46.38 (6.25)
S03	35.18 (1.25)	35.55 (1.06)	2.95 (0.08)	3.00 (0.08)	58.41 (6.86)	51.90 (7.47)
S04	41.11 (1.22)	41.68 (1.17)	2.81 (0.07)	2.82 (0.05)	56.97 (8.71)	50.55 (7.60)
S05	36.38 (2.08)	38.24 (4.36)	3.21 (0.10)	3.12 (0.08)	68.77 (7.65)	64.86 (7.44)
S06	35.12 (3.05)	34.30 (2.63)	3.01 (0.09)	3.07 (0.08)	48.56 (5.13)	53.81 (5.57)
S07	39.24 (1.92)	40.85 (2.37)	2.99 (0.08)	2.98 (0.12)	58.06 (8.37)	51.73 (6.10)
S08	39.45 (1.99)	39.58 (1.65)	3.02 (0.08)	3.02 (0.07)	47.92 (7.23)	44.88 (6.11)
Mean	39.36 (3.72)	39.72 (3.59)	2.97 (0.13)	2.97 (0.10)	54.23 (7.86)	51.20 (6.44)
*p*-value		0.31		0.79		0.08

*These estimates were obtained by training and evaluating on a single subject. Outcomes are averaged over approximately 400 steps (left and right combined). P-values are calculated using a paired t-test with the subject mean values*.

#### 3.1.2. Kinetics estimation

Table 4 shows an overview of performance when different combinations of input features (joint angles, pelvis and lower leg accelerations) are evaluated. On average the best results (marked in bold for individual subjects) were achieved using a combination of all vertical accelerations and joint angles as input features. Therefore, results presented below are obtained when ANN_2_ was trained using these features.

**Table 4:** Accuracy of the estimated vertical ground reaction force (vGRF) using different input features (namely: joint angles (θ_*joint*_), pelvis vertical acceleration (*a*_*P*_), all (pelvis, left and right lower leg) vertical accelerations (*a*_*P*+*L*_) or a combination of these).

**Subjects**		**Features**									
		***a*_*P*_**		***a*_*P*+*L*_**		**θ_*joint*_**		***a*_*P*_ & θ_*joint*_**		***a*_*P*+*L*_ & θ_*joint*_**	
		**ρ**	**RMSE (σ)**	**ρ**	**RMSE (σ)**	**ρ**	**RMSE (σ)**	**ρ**	**RMSE (σ)**	**ρ**	**RMSE (σ)**
S01	L vGRF	0.97	0.26 (0.03)	0.98	0.21 (0.03)	0.99	0.20 (0.06)	0.99	0.15 (0.04)	**0.99**	**0.12 (0.03)**
	R vGRF	0.96	0.26 (0.02)	0.99	0.17 (0.02)	0.99	0.21 (0.05)	0.99	0.16 (0.04)	**0.99**	**0.15 (0.04)**
S02	L vGRF	0.94	0.33 (0.03)	0.97	**0.23 (0.02)**	**0.98**	0.24 (0.07)	0.97	0.24 (0.05)	0.97	0.25 (0.03)
	R vGRF	0.97	0.27 (0.03)	**0.97**	**0.25 (0.03)**	0.96	0.29 (0.08)	0.96	0.28 (0.06)	0.97	0.25 (0.04)
S03	L vGRF	0.96	0.29 (0.03)	0.99	0.14 (0.03)	0.99	0.20 (0.07)	0.99	0.12 (0.04)	**0.99**	**0.10 (0.03)**
	R vGRF	0.95	0.32 (0.03)	0.99	0.11 (0.03)	0.99	0.16 (0.05)	0.99	0.10 (0.03)	**0.99**	**0.09 (0.02)**
S04	L vGRF	0.96	0.25 (0.03)	**0.99**	**0.14 (0.03)**	0.99	0.16 (0.05)	0.99	0.17 (0.04)	0.99	0.15 (0.04)
	R vGRF	0.96	0.27 (0.03)	0.99	0.13 (0.04)	0.99	0.20 (0.07)	0.99	0.13 (0.04)	**0.99**	**0.11 (0.03)**
S05	L vGRF	0.97	0.28 (0.04)	**0.98**	**0.25 (0.05)**	0.95	0.37 (0.11)	0.97	0.30 (0.07)	0.97	0.30 (0.07)
	R vGRF	0.98	0.27 (0.03)	**0.98**	**0.25 (0.05)**	0.93	0.44 (0.14)	0.96	0.33 (0.08)	0.96	0.33 (0.08)
S06	L vGRF	0.95	0.32 (0.03)	**0.98**	**0.22 (0.04)**	0.95	0.38 (0.09)	0.96	0.34 (0.07)	0.96	0.30 (0.05)
	R vGRF	0.94	0.35 (0.04)	**0.98**	**0.21 (0.04)**	0.93	0.42 (0.10)	0.95	0.36 (0.06)	0.95	0.33 (0.05)
S07	L vGRF	0.91	0.40 (0.30)	**0.96**	**0.27 (0.04)**	0.93	0.38 (0.10)	0.96	0.30 (0.06)	0.96	0.28 (0.05)
	R vGRF	0.93	0.38 (0.03)	0.96	0.28 (0.04)	0.96	0.33 (0.08)	0.96	0.29 (0.07)	**0.97**	**0.25 (0.06)**
S08	L vGRF	0.97	0.27 (0.02)	0.99	0.12 (0.02)	0.99	0.20 (0.07)	0.99	0.14 (0.04)	**0.99**	**0.11 (0.03)**
	R vGRF	0.96	0.28 (0.03)	0.99	0.14 (0.03)	0.98	0.24 (0.07)	0.99	0.19 (0.05)	**0.99**	**0.12 (0.04)**

*The evaluated set of features is shown above each column. These results were obtained using single subject training and evaluation. Pearson's correlation coefficient (ρ) is calculated for each contact and averaged over approximately 200 stance phases for each subject (S01, S02, S03, S04, S05, S06, S07 and S08). The Root Mean Squared Error (RMSE) is calculated similarly over all contacts, and the standard deviation of the RMSE is shown in brackets. The highest correlations (ρ) and smallest RMSE are shown in bold*.

**Figure 5:**
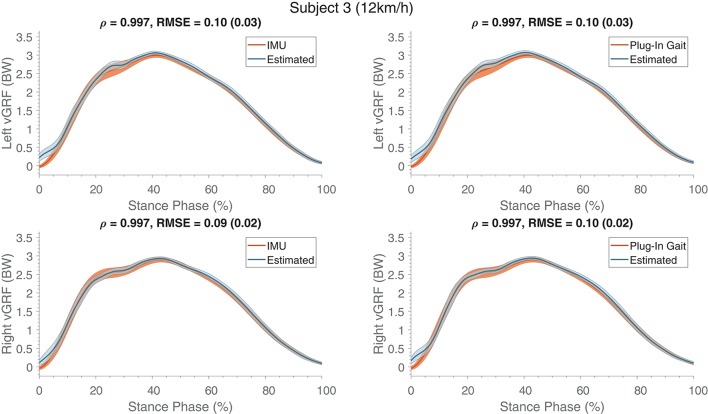
Mean (and standard deviation band) of the estimated ground reaction forces (in BW) are presented (normalized to the stance phase) compared to their respective references (IMU and Plug-In Gait joint angle output). These estimates were obtained from training and evaluating on a single subject, similar results were obtained for the other subjects. The **top** row shows the forces of the left contacts and the **bottom** row presents the right contacts. At the top of each graph Pearson's correlation coefficient, root mean square error (RMSE) and the standard deviation (between the brackets) are specified, which were calculated for the estimate compared to its respective reference kinematics.

The estimated ground reaction profiles of a representative subject (S03) are shown in Figure 5 for ANN_2_ based on both reference kinematics (IMUs and Plug-In Gait). Similarly to what was observed for the estimated knee F/E angles, differences between the networks (ANN_2_) trained on the various references are minimal. Largest differences between the estimated and reference vGRF can be seen at the beginning of stance phase. However, peak values are estimated with high accuracy, resulting in correlation coefficients larger than 0.96.

Results for the discrete outcomes (peak vGRF and loading rate) can be found in Table 3. Mean peak vGRF differences between the estimate and its reference are within 0.09 BW for all subjects, which resulted in no significant differences (*p* > 0.05). Variation between the estimate and its reference is larger for the loading rate, however this difference is still not significant (*p* > 0.05). Figures 4B,C show the Bland-Altman plots for both the peak vGRF and loading rate. A small bias of 0.01 BW is present in the estimated peak vGRF, with limits of agreement –0.17 to 0.18 BW. The loading rate is estimated with a bias of –2.9 BW/s with limits of agreement –16 to 10 BW/s. Both plots show occasional outliers for multiple subjects.

#### 3.1.3. Variation in running speeds

**Figure 6:**
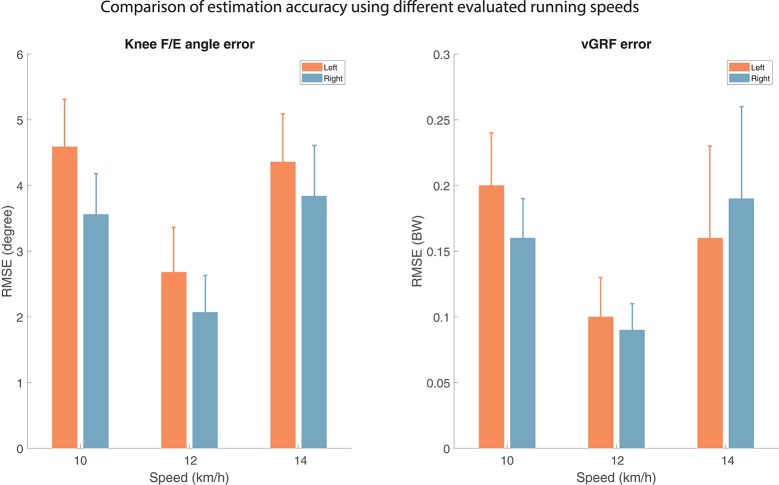
Accuracy of the estimated vertical ground reaction force (vGRF) and knee flexion/extension (F/E) angle for different evaluated speeds, hence the other speeds are part of the training dataset, using single subject training and evaluation, as described in section 2.4. The artificial neural networks were trained with and evaluated relative to a full-body inertial kinematic measurement (Table 1, training scheme 1). The results for a representative subject are shown in this graph. The Root Mean Squared Error (RMSE) is calculated over all stride/stance phases and averaged over approximately 200 strides for each different evaluated speed (10, 12, and 14 km/h).

Extrapolation capabilities of the proposed approach were investigated by evaluating different running speeds for subject 3. Figure 6 shows RMSEs for the evaluated speeds, where the remaining trials are in the training dataset. This figure shows that the most accurate continuous estimation can be achieve when an intermediate speed (12 km/h) is used, rather than the ones which are slower (10 km/h) or faster (14 km/h) than those in their respective training datasets.

Additionally, discrete outcome measures were evaluated for the same subject, which are presented in Table 5. The peak vGRF and maximal knee flexion during stance also show that interpolating speeds results in more accurate outcomes than extrapolating. However, this trend is not present for the loading rate accuracy.

**Table 5:** The variation in discrete outcome measures for different speeds in subject 3.

**Parameter**	**Speed**	**Reference (IMU)**		**Estimate**	
		**Left**	**Right**	**Left**	**Right**
Max knee flexion (degrees)	10 km/h	34.49 (1.10)	33.90 (1.20)	30.57 (0.98)	30.24 (1.22)
	12 km/h	35.64 (1.20)	34.71 (1.13)	36.18 (0.71)	34.92 (0.98)
	14 km/h	36.93 (1.24)	35.11 (1.16)	38.22 (1.58)	36.23 (2.76)
peak vGRF (BW)	10 km/h	2.85 (0.06)	2.77 (0.06)	2.76 (0.07)	2.67 (0.08)
	12 km/h	3.00 (0.06)	2.90 (0.07)	3.06 (0.04)	2.93 (0.05)
	14 km/h	3.13 (0.07)	3.00 (0.07)	2.96 (0.13)	2.92 (0.10)
Loading rate (BW/s)	10 km/h	52.92 (5.82)	55.11 (6.10)	46.05 (5.92)	56.05 (8.37)
	12 km/h	55.47 (6.12)	61.34 (6.29)	47.67 (6.28)	56.13 (6.03)
	14 km/h	63.25 (7.31)	67.17 (9.78)	59.11 (9.06)	55.52 (13.91)

*The mean (and standard deviation) of peak vGRF, loading rate and max knee flexion during stance are shown for both the estimate and its corresponding reference (based on inertial full-body motion capture data), these are calculated over approximately 400 steps (left and right combined). The artificial neural networks were trained using running data of two speeds (different from the evaluation speed), while the shown speed was used for evaluation*.

### 3.2. Multiple subject learning

**Figure 7:**
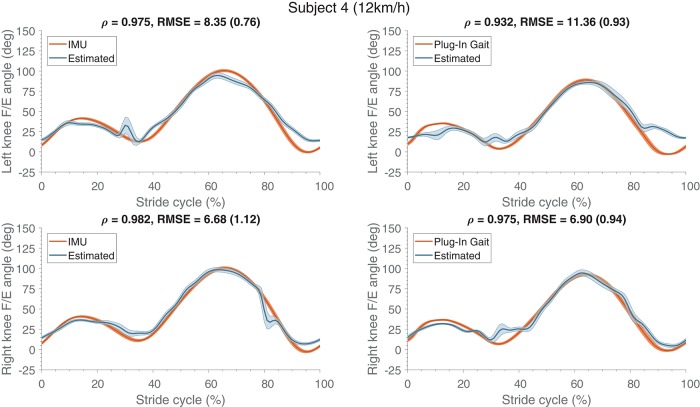
Mean (and standard deviation band) of the flexion/extension knee joint angle (in degrees) estimates are presented (normalized to the stride cycle) compared to their respective references (IMU and Plug-In Gait joint angle output). These estimates were obtained from training on multiple subjects and evaluating on a different subject, and were comparable to the other evaluated subjects. The **top** row shows the angles of the left side and the **bottom** row presents the right side. At the top of each graph Pearson's correlation coefficient, root mean square error (RMSE) and the standard deviation (between the brackets) are specified, which were calculated for the estimate and its respective reference kinematics.

The generalization performance of both ANNs were evaluated by training with all different combinations of subjects in the training and evaluation datasets. Table 6 (top-half) shows the results of kinematics for the different evaluated subjects. Seven out of the eight subject show correlations larger than 0.9, indicating good agreement. However, the RMSE is expectantly larger than for single subject learning (section 3.1). The estimated knee F/E angles for subjects 1 and 3 are significantly less accurate. Additionally, the mean estimated knee F/E angle profiles of subject 4 are shown in Figure 7, with the measured references used for comparison. The stance phase (until approximately 30% of the stride cycle) is estimated with higher accuracy than the swing phase, same behavior can be seen for single subject learning (Figure 3).

**Table 6:** Accuracy of the estimated knee flexion/extension (F/E) angles (by ANN_1_) and vertical ground reaction forces (vGRF) (by ANN_2_) using different training outputs (namely: IMU or Plug-in Gait-based) by training on data of all subjects except for one which is used for the evaluation at 12 km/h.

**Knee F/E angle accuracy**								
**Subjects**	**IMU**				**Plug-in Gait**			
	**Left F/E**		**Right F/E**		**Left F/E**		**Right F/E**	
	**ρ**	**RMSE (σ)**	**ρ**	**RMSE (σ)**	**ρ**	**RMSE (σ)**	**ρ**	**RMSE (σ)**
S01	0.88	19.11 (4.92)	0.83	19.47 (3.66)	0.77	25.05 (2.20)	0.83	23.57 (2.13)
S02	0.99	8.57 (0.74)	0.99	8.09 (0.79)	0.98	11.87 (1.08)	0.98	6.76 (0.71)
S03	0.95	14.92 (1.54)	0.94	11.08 (1.32)	0.91	15.19 (1.54)	0.91	22.57 (4.03)
S04	0.98	8.35 (0.76)	0.98	6.68 (1.12)	0.93	11.36 (0.93)	0.98	6.90 (0.94)
S05	0.98	9.89 (1.10)	0.98	7.00 (1.28)	0.96	19.62 (3.62)	0.97	7.41 (1.43)
S06	0.98	7.33 (1.00)	0.99	6.99 (1.07)	0.97	7.70 (0.99)	0.98	8.76 (1.46)
S07	0.98	5.88 (0.68)	0.99	4.83 (0.99)	0.98	6.83 (0.81)	0.98	6.62 (0.85)
S08	0.98	6.36 (1.18)	0.99	4.66 (0.92)	0.98	6.29 (1.09)	0.99	7.21 (0.89)
**vGRF accuracy**								
**Subjects**	**IMU**				**Plug-in Gait**			
	**Left vGRF**		**Right vGRF**		**Left GRF**		**Right vGRF**	
	**ρ**	**RMSE (σ)**	**ρ**	**RMSE (σ)**	**ρ**	**RMSE (σ)**	**ρ**	**RMSE (σ)**
S01	0.92	0.45 (0.10)	0.90	1.25 (0.25)	0.94	0.52 (0.11)	0.86	0.56 (0.08)
S02	0.95	0.31 (0.08)	0.99	0.16 (0.03)	0.98	0.22 (0.04)	0.97	0.27 (0.05)
S03	0.95	0.50 (0.18)	0.98	0.60 (0.09)	0.83	0.81 (0.19)	0.97	0.38 (0.07)
S04	0.98	0.26 (0.06)	0.95	0.32 (0.05)	0.95	0.34 (0.19)	0.98	0.30 (0.05)
S05	0.99	0.21 (0.05)	0.97	0.32 (0.07)	0.97	0.33 (0.08)	0.99	0.20 (0.07)
S06	0.98	0.25 (0.04)	0.94	0.36 (0.03)	0.97	0.28 (0.04)	0.99	0.20 (0.04)
S07	0.96	0.30 (0.04)	0.98	0.22 (0.04)	0.97	0.29 (0.05)	0.97	0.27 (0.05)
S08	0.93	0.46 (0.05)	0.98	0.24 (0.04)	0.91	0.44 (0.05)	0.98	0.28 (0.04)

*Pearson's correlation coefficient (ρ) is calculated for each stride and averaged over approximately 200 strides for each different test subject (S01, S02, S03, S04, S05, S06, S07 and S08). The Root Mean Squared Error (RMSE) is calculated similarly over all strides*.

**Figure 8:**
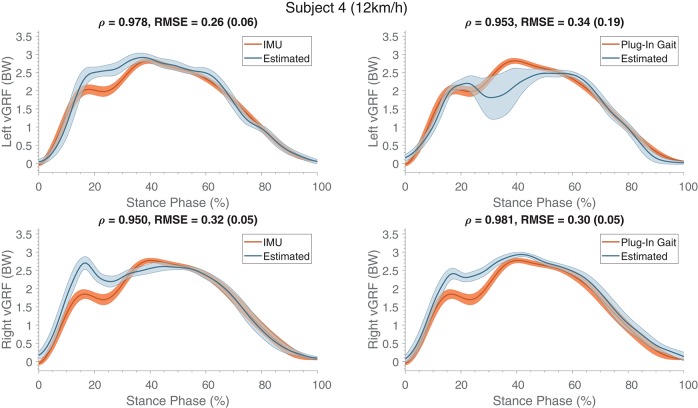
Mean (and standard deviation band) of the estimated vertical ground reaction forces (in BW) are presented (normalized to the stance phase) compared to the measured reference. These estimates were obtained from training on multiple subjects and evaluating on a different subject, and were comparable to the other evaluated subjects. The **top** row shows the forces of the left contacts and the **bottom** row presents the right contacts. At the top of each graph Pearson's correlation coefficient, root mean square error (RMSE) and the standard deviation (between the brackets) are specified, which were calculated for the estimate and its respective reference kinematics.

Results of the kinetic estimations can be seen in Table 6 (bottom-half). Similar to the joint angles, vGRFs are mostly estimated with correlations larger than 0.9 indicating good agreement with the measurements. However, subjects 1 and 3 show lower correlation coefficients, as was also seen for the kinematics. Vertical ground reaction force profiles of one representative subject (S04) are shown in Figure 8, which shows an increase in RMSEs compared to the single subject learning (Figure 5). The maximum estimated ground reaction forces are mostly comparable to the reference.

The accuracy of estimating discrete outcome measures is shown in Table 7. The estimation accuracy varies between different subjects and outcome measures. However, in most cases an increase in error can be seen when comparing to the single subject training (Table 3). Additionally, an increase in the standard deviations of the different estimated outcome measures can be seen. However, the estimated outcome measures and the corresponding references were not found to be significantly different.

**Table 7:** The mean (and standard deviation) of discrete outcome measures for both the estimate and its corresponding reference (based on inertial full-body motion capture data) of all subjects.

**Subjects**	**Max knee F/E angle (degrees)**		**vGRF peak (BW)**		**Loading rate (BW/s)**	
	**Reference**	**Estimate**	**Reference**	**Estimate**	**Reference**	**Estimate**
S01	45.41 (3.56)	65.90 (24.61)	2.79 (0.08)	3.82 (0.96)	44.39 (7.37)	33.86 (35.59)
S02	42.96 (1.55)	33.04 (1.19)	2.96 (0.07)	2.92 (0.13)	50.72 (4.93)	43.91 (7.38)
S03	35.18 (1.25)	40.21 (5.38)	2.95 (0.08)	2.72 (0.64)	58.41 (6.86)	38.72 (8.27)
S04	41.11 (1.22)	38.35 (3.73)	2.81 (0.07)	2.86 (0.14)	56.97 (8.71)	70.75 (12.11)
S05	36.38 (2.08)	41.88 (3.81)	3.21 (0.10)	3.15 (0.19)	68.77 (7.65)	48.87 (17.20)
S06	35.12 (3.05)	37.42 (2.68)	3.01 (0.09)	3.01 (0.12)	48.56 (5.13)	41.47 (3.58)
S07	39.24 (1.92)	38.97 (3.65)	2.99 (0.08)	2.91 (0.08)	58.06 (8.37)	58.19 (8.75)
S08	39.45 (1.99)	38.36 (1.68)	3.02 (0.08)	3.17 (0.23)	47.92 (7.23)	56.06 (10.79)
Mean	39.36 (3.72)	41.77 (10.08)	2.97 (0.13)	3.07 (0.34)	54.23 (7.86)	48.98 (12.10)
*p*-value		0.47		0.26		0.37

*These estimates were obtained by training on multiple subjects and evaluating on a different subject (using running data at 12 km/h). Outcomes are averaged over approximately 400 steps (left and right combined). P-values are calculated using a paired t-test with the subject mean values*.

## 4. Discussion

This work shows that sagittal knee kinematics and vGRF can be estimated using only three inertial sensors placed on the lower legs and pelvis, in particular, the peak vGRF, maximal knee F/E angles during stance, and the knee F/E angles and vGRF profiles are estimated with no significant differences with respect to the reference.

Estimation of joint angles for a single subject has shown to be more accurate (average RMSE < 5°) than was achieved in previous work of the authors (average RMSE ≈7°) (Wouda et al., 2016). This can partly be explained by the difference in composition of the training databases between both methods, since the current dataset had less variation of motions, i.e., only running. This approach requires obtaining reference kinetics and kinematics of each subject, i.e., each subject has to run on an instrumented treadmill.

Additionally, multiple subject learning results showed good agreement (ρ > 0.9) for most subjects in the continuous outcomes. However, the ANNs could not generalize over all idiosyncrasies of the individual subjects as RMSEs and differences in discrete outcomes increased, expectantly. Subjects had different landing patterns (heel, mid, or forefoot striking), which may be a reason for the degraded performance shown for example in subject 1. By including more subjects different models could be trained for each different landing phenotype. Alternatively, larger soft-tissue artifacts of the inertial sensors compared to the other subjects may explain the degraded performance.

No significant differences were found between any of the reference and estimated discrete outcome measures, for both evaluation scenarios. However, the required accuracy would largely be defined by the application of interest. An example of such an application could be tracking kinematic/kinetic changes due to fatigue, since they may relate to increased chance of injury (Reenalda et al., 2016). However, more data (specific for such an application, e.g., running under fatigue) should be acquired to evaluate if the proposed approach can track such differences.

The running mechanics in this work are estimated based on inertial or optical motion capture data. Each of these technologies have their advantages and disadvantages (Field et al., 2011). Differences in the reference knee F/E profiles for the different technologies are observed for the results in section 3.1.1, which can be explained by differences in the underlying models of the human body and their assumptions (Kainz et al., 2016). However, the estimated kinematics based on the different technologies are similar to their respective measured kinematics. This shows that the method has potential to be applied in this context irrespective of the preferred technology for recording training data. Therefore, the proposed method has potential to estimate output based on other kinematic references, such as biomechanical models driven by optical data (Delp et al., 2007; Stief et al., 2013).

The measured dataset contains only treadmill running, however, the proposed method is not limited to be applied under these conditions. Evaluating the proposed method in a different setting (e.g., outdoor running) might result in less accurate estimations of knee F/E angles and vGRFs. To improve such results, the dataset can be extended by including running at different slopes of the treadmill. Furthermore, 3D ground reaction forces could be measured using pressure insoles for example (Rouhani et al., 2010), which enables data collection in any running environment for training data collection. Extrapolating kinematic and kinetic data outside of the training dataset appears to be more difficult than interpolating such data. This was shown by the degraded performance after training with different running speeds or extrapolating over various subjects. This indicates that careful construction of the training dataset is required to obtain the best possible performance.

A limitation of the proposed method is that only vertical kinetics can be estimated. This can be contributed to the available measurement setup, since it would require a treadmill instrumented with a force plate that can measure three-dimensional forces. However, our proposed method could be extended using the three-dimensional GRF estimation approach of Karatsidis et al. (2017) using full-body inertial motion capture. Furthermore, only sagittal plane knee kinematics could be estimated in the proposed approach, possibilities of estimating kinematics of other joints and/or planes would require additional research.

The concatenated ANN approach allows for training the ANN_1_ (kinematics) independent of the ANN_2_ (kinetics). This enables the use of only inertial motion capture data in various environments for training ANN_1_. Instead of concatenating two ANNs, a single ANN could be trained to map relative orientations and vertical accelerations to ground reaction forces and joint angles. Initial tests show comparable results for single subject training, however, multiple subject training was less successful. When one ANN is trained to estimate both kinematics and kinetics, cross-dependencies between features and outputs become important, which is less so for concatenated ANNs. This can be seen in the differences in accuracy between estimation of kinematics (ANN_1_) and kinetics (ANN_2_) for multiple subject training in section 3.2.

Figure 5 shows differences in the measured reference vGRF between left and right stance phases, which can also be seen from the estimated output. This could indicate that the proposed method is capable of detecting differences between left and right kinetics. Note that, given the relatively short duration of the running sessions, effects of fatigue could not be evaluated using the current setup, but it is an interesting future development.

The estimated vertical ground reaction forces (ρ > 0.99 and RMSE < 0.27 BW) using the proposed method are comparable to that of Faber et al. (2016) (*R*^2^ > 0.981 and RSME < 10 N), who estimated GRFs during a bending task by using a full-body inertial motion capture system. Karatsidis et al. (2017) evaluated a similar approach on walking using inertial sensors, where the errors are comparable to the ones reported in the proposed method. Charry et al. (2013) showed that by exploiting only tibial accelerations to estimate peak vGRFs an approximate RMSE of 6% can be achieved, however this method was only applied to training and testing on individual subjects. Shippen and May (2012) estimated vGRF more accurately (3% error) than the proposed method, by relying on full-body optical motion capture for their method. Pavei et al. (2017) reported similar performance in estimation of the loading rate, while our proposed method was shown to estimate peak vGRFs more accurately. Charry et al. (2013) reported peak vGRF estimation errors of approximately 6%, whereas our proposed method is able to estimate peak vGRF with an accuracy of <0.10 BW (≈3.5%).

## 5. Conclusions

This work has shown the potential of estimating kinetics (vGRF) and kinematics (knee F/E angles) during running using a minimal on-body sensor setup (namely, three sensor devices placed on the lower legs and pelvis). Best performance can be obtained when the proposed approach is applied to a single subject. Training over multiple subjects was shown to be possible, since good agreement between the estimates and references were achieved, however the RMSEs are larger than for single subject training. In other words, the proposed method has potential to be applied for individual subjects, and with additional research can be extended for running in various environments.

## Author contributions

The study design was conceptualized by FW, MG, GB, EM, and JR. The data collection was conducted by FW and EM. The data was analyzed by FW under the supervision of all authors. The manuscript was drafted by FW, and all authors contributed significantly to revisions, literature review and the discussion of results. All authors approved the final version and agreed to be accountable for all aspects of this work.

### Conflict of interest statement

MG and GB are employed by Xsens Technologies BV. The other authors declare that the research was conducted in the absence of any commercial or financial relationships that could be construed as a potential conflict of interest.

## Footnotes

^1^https://www.vicon.com/downloads/documentation/plug-in-gait-product-guide
